# AAV2-mediated gene therapy for Bietti crystalline dystrophy provides functional CYP4V2 in multiple relevant cell models

**DOI:** 10.1038/s41598-022-12210-8

**Published:** 2022-06-09

**Authors:** Jiang-Hui Wang, Grace E. Lidgerwood, Maciej Daniszewski, Monica L. Hu, Georgina E. Roberts, Raymond C. B. Wong, Sandy S. C. Hung, Michelle E. McClements, Alex W. Hewitt, Alice Pébay, Doron G. Hickey, Thomas L. Edwards

**Affiliations:** 1 Centre for Eye Research Australia, Royal Victorian Eye and Ear Hospital, Level 7, 32 Gisborne Street, East Melbourne, VIC 3002 Australia; 2 Department of Anatomy and Physiology, The University of Melbourne, Parkville, Australia; 3 Ophthalmology, Department of Surgery, The University of Melbourne, East Melbourne, VIC Australia; 4 Shenzhen Eye Hospital, Shenzhen University School of Medicine, Shenzhen, China; 5 Department of Clinical Neurosciences, Nuffield Laboratory of Ophthalmology, University of Oxford, Oxford, UK; 6 Menzies Institute for Medical Research, University of Tasmania, Hobart, TAS Australia; 7 Department of Surgery, Royal Melbourne Hospital, The University of Melbourne, Parkville, Australia

**Keywords:** Eye diseases, Medical research

## Abstract

Bietti crystalline dystrophy (BCD) is an inherited retinal disease (IRD) caused by mutations in the *CYP4V2* gene. It is a relatively common cause of IRD in east Asia. A number of features of this disease make it highly amenable to gene supplementation therapy. This study aims to validate a series of essential precursor in vitro experiments prior to developing a clinical gene therapy for BCD. We demonstrated that HEK293, ARPE19, and patient induced pluripotent stem cell (iPSC)-derived RPE cells transduced with AAV2 vectors encoding codon optimization of *CYP4V2* (AAV2.coCYP4V2) resulted in elevated protein expression levels of CYP4V2 compared to those transduced with AAV2 vectors encoding wild type *CYP4V2* (AAV2.wtCYP4V2), as assessed by immunocytochemistry and western blot. Similarly, we observed significantly increased CYP4V2 enzyme activity in cells transduced with AAV2.coCYP4V2 compared to those transduced with AAV2.wtCYP4V2. We also showed CYP4V2 expression in human RPE/choroid explants transduced with AAV2.coCYP4V2 compared to those transduced with AAV2.wtCYP4V2. These preclinical data support the further development of a gene supplementation therapy for a currently untreatable blinding condition—BCD. Codon-optimized *CYP4V2* transgene was superior to wild type in terms of protein expression and enzyme activity. Ex vivo culture of human RPE cells provided an effective approach to test AAV-mediated transgene delivery.

## Introduction

Bietti crystalline dystrophy (BCD, OMIM #210370) is a progressive, rare, autosomal recessive inherited retinal disease (IRD)^1^. The clinical phenotype of BCD is characterised by numerous distinctive glistening yellow-white crystalline deposits primarily in the retinal pigment epithelium (RPE) of the macula. Patients typically develop nyctalopia, paracentral visual field loss and loss of central acuity, progressing to legal blindness by the fifth or sixth decade of life^2^. BCD is rare in Western countries but is more common in East Asia, particularly in Chinese and Japanese populations^3,4^. The predicted prevalence of BCD is estimated to be 1 in 67,000 individuals, therefore affecting an estimated 21,000 patients in China and 5,000 in the USA^3,5^. Currently there is no treatment available for BCD.

BCD is caused by mutations in the cytochrome P450 (CYP) family 4 subfamily V polypeptide 2 gene (*CYP4V2*)^6^, which is involved in the metabolism of endogenous fatty acids and steroids through ω-hydroxylation^7^. Over 57 unique mutations of *CYP4V2* are known to cause BCD, most of which are missense or nonsense mutations^8^. The most common *CYP4V2* mutation is c.802-8_810del17insGC, which leads to deletion of exon 7^9,10^. CYP4V2 is an orphan member of the cytochrome P450 family. Its expression is enriched in retinal pigmented epithelial (RPE) cells^2^. Cytochrome P450 enzymes are involved in the metabolism of fatty acids and steroids through ω-hydroxylation and function together with mitochondrial and peroxisomal *α/β*-oxidation enzymes to degrade cellular lipids^11^. Although abnormal lipid metabolism has been identified in circulating lymphocytes, serum and skin fibroblasts of patients with BCD^8,12^, the disease phenotype seems to be restricted to the eye. An early biochemical tracer study suggested a cellular defect in the anabolism of ω-3-polyunsaturated fatty acids in lymphocytes and fibroblasts from patients with BCD^13^. Altered serum lipid profiles have also been noted in the plasma of patients with BCD, suggesting a defect in the synthesis of oleic acid^12^. Moreover, a recent study showed that free cholesterol is accumulated in induced pluripotent stem cells (iPS)-derived RPE cells from BCD patients, likely due to lysosomal dysfunction and impaired autophagy, suggesting local abnormal cholesterol metabolism may play a role in pathogenesis of BCD^14^. The precise composition of the BCD crystals remains elusive.

The clinical characteristics of BCD, together with its underlying biology, make this disease an ideal candidate for retinal gene augmentation therapy. First, the striking crystalline fundus phenotype is unique and so facilitates early targeted genetic testing of most affected patients. Second, the disease is caused by biallelic loss of function mutations in *CYP4V2*, supporting a gene augmentation strategy. Third, the coding sequence of *CYP4V2* (1578 bp) is within the packaging capacity of adeno-associated virus (AAV) – the most established viral vector for clinical gene therapy^15–17^. Fourth, the primary pathology of BCD occurs in RPE cells that can be directly targeted by AAV through subretinal injection. Fifth, there is a wide therapeutic window between symptom onset and significant vision impairment that offers opportunities for treatment.

In the present study, we evaluated viral-mediated expression and function of CYP4V2 in different cell lines transduced with an AAV2 vector encoding codon optimized human *CYP4V2* (AAV2.coCYP4V2). The cellular models included HEK293 cells, ARPE19 cells (a commercially available human RPE cell line), and BCD induced pluripotent stem cells (iPSCs)-derived RPE cells. In addition, we demonstrated ex vivo transduction of human retinal explants, arguably the most suitable model for retinal translational research, with AAV2.coCYP4V2.

## Materials and methods

### Ethics approval

Ethics approval for collection of patient samples was obtained from the institutional review board of the University of Melbourne (1545484, 1545394) and the Royal Victorian Eye and Ear Hospital (11/1031H & HREC13/1151H). Written informed consent was obtained from all human participants after discussion of the procedures and alternatives as well as potential benefits and risks. All clinical investigations were conducted according to the WMA Declaration of Helsinki. Post-mortem eyes were collected by the Lions Eye Donation Service (Royal Victorian Eye and Ear Hospital).

### Human iPSC generation and maintenance

The cohort included the iPSC line ES4CL2 (a gift from Prof. Jamie Thomson, University of Wisconsin) and the BCD iPSC line MBE01784 generated in-house. BCD patients’ fibroblasts were reprogrammed to iPSCs by nucleofection of episomal vectors containing *OCT-4*, *SOX2*, *KLF4*, *L-MYC*, *LIN28*, and shRNA against *p53*, and reprogrammed colonies were expanded and characterised. The iPSC line (foreskin) 4 clone 2^18^ was used as a control. MBE01784 was generated using skin fibroblasts obtained from a 42 year-old patient with BCD, as previously described^19,20^. Briefly, fibroblasts at passage number 2 were nucleofected (Lonza Amaxa Nucleofector) with episomal vectors expressing OCT-4, SOX2, KLF4, L-MYC, LIN28 and shRNA against p53^21^ in feeder- and serum-free conditions using TeSR™-E7™ medium (Stem Cell Technologies). The reprogrammed iPSCs were selected as a polyclonal line using an automated platform as previously described^19^, subsequently expanded and characterised. All iPSC lines were maintained manually onto vitronectin XF™-coated plates (Stem Cell Technologies) in StemFlex™ (Thermo Fisher Scientific), with media changes every second day and weekly passaging using ReLeSR™ (Stem Cell Technologies). Fibroblasts and iPSCs were mycoplasma-free (MycoAlert mycoplasma detection kit, Lonza, Switzerland). Immunocytochemistry was used to examine iPSC pluripotency with OCT-4 and TRA-1-60. Cells were differentiated to the three-germ layer. Embryoid bodies were obtained following standard protocols as previously described^19^. Germ layer differentiation was assessed by immunocytochemistry using NESTIN, AFP and SMA antibodies.

### Differentiation of iPSCs into RPE cells

Differentiation of iPSCs into RPE cells was performed as we previously described^22^. iPSCs were differentiated in E6 medium (Stem Cell Technologies), supplemented with N2, penicillin—streptomycin (Life Technologies) for 28–32 days and subsequently switched to RPE medium (αMEM, 5% FBS, non-essential amino acids, N1, penicillin—streptomycin—glutamate, taurine—hydrocortisone—triiodothyronine) and cultured for further 28 days with a medium change every second day. Cells were passaged with 0.25% trypsin–EDTA and plated onto growth factor-reduced Matrigel-coated plates (Corning) for 76–88 days.

### HEK293 and ARPE19 Cell Culture and Transfection

HEK293 and ARPE19 cells were cultured in Dulbecco’s modified Eagle’s medium (DMEM) (11965118; Gibco) and DMEM/F-12 (11320033; Gibco), respectively, supplemented with 10% FBS (10099141; Gibco) and 1% penicillin/streptomycin (15140122; Gibco). Fibroblast cells were cultured in media used for HEK293 cells. All cells were grown at 37 °C with 5% CO_2_. Plasmid transfection was performed using lipofectamine 3000 (L3000008; Life Technologies), with 1.0 µg, 0.2 µg and 40 ng of plasmids per well of a 6-well, 24-well and 96-well plates, respectively, according to the manufacturer’s instructions.

### Transcriptome analysis using RNA-seq

Total RNA was extracted using a RNeasy kit and treated with DNase 1 (Qiagen). Three donor samples of human RPE/choroid were processed for RNA-seq^23^. RNA-seq was performed by the Australian Genome Research Facility. Briefly, RNA quality was assessed using Bioanalyzer, and transcriptome libraries were prepared using the TruSeq Stranded mRNA kit (Illumina). Subsequently, samples were processed for 100 bp single-end sequencing by using the Illumina Novaseq 6000 and yielded ~ 38–50 million reads per sample. Adapter sequences were removed from the raw fastq files of single end reads, and the low-quality reads were dropped by Trimgalore 0.6.7 (Babraham Bioinformatics, Cambridge, UK). Filtering parameters were set as follows: -q 25 -length 50 -e 0.1 -stringency 5. The trimmed reads were subjected to alignment with default settings using HISAT2-2.2.1. The human reference genome was hg38. Aligned RNA-seq data were counted over gene exons using featureCounts 2.0.1. Genes were annotated as per the gencode.v38 annotation file. The transcriptome data of the human RPE/choroid in this study are available in the NCBI Gene Expression Omnibus database (GSE181550).

### Immunocytochemistry and immunohistochemistry

Samples of RPE/choroid explants were fixed in 4% paraformaldehyde (PFA) in PBS, pH 7.4, for 1 h and then subjected to 30% sucrose cryoprotection. The following day they were embedded in Tissue-Tek O.C.T. Compound (#4583, Sakura Finetek). Sections (10 µm) were cut on a cryostat and mounted on glass slides (SuperFrost Plus; Menzel-Gläser, Braunschweig, Germany). Cells on a coverslip were fixed in 4% PFA in PBS, followed by PBS wash. Immunocytochemistry was performed using the following primary antibodies at 4 °C for overnight incubation: mouse anti-OCT3/4 (2.5 μg/ml, sc-5279, Santa Cruz Biotechnology), mouse anti-TRA-1-60 (1 μg/ml, MA1-023-PE, Thermo Fisher Scientific), mouse anti-NESTIN (10 μg/ml, AB22035, Abcam), rabbit anti-alpha-fetoprotein (AFP, 10 μg/ml, st1673, Sigma-Aldrich), mouse anti-smooth muscle actin (SMA, 10 μg/ml, MAB1420, R&D Systems), mouse anti-CYP4V2 (1:400, ab69392, Abcam), rabbit anti-CYP4V2 (1:500, SAB1410565, Sigma) and mouse anti-RPE65 (1:400, ab13826, Abcam). Cells or frozen sections were then immunostained with isotype-specific secondary antibodies (Alexa Fluor 568 or 488, Life Technologies). Nuclei were counterstained using DAPI (Sigma-Aldrich). Specificity of the staining was verified by the absence of staining in negative controls consisting of the appropriate negative control immunoglobulin fraction (DAKO). Images were acquired on Leica SP5 confocal microscope using Leica Application Software or Nikon C1 confocal laser scanning microscope.

### Quantitative real time polymerase chain reaction (qRT-PCR)

Total RNA was isolated with a miRNeasy mini kit (217084; Qiagen). The quantity of RNA was measured by spectrophotometry. Total RNA (100 ng) was reversely transcribed to cDNA using a high-capacity reverse transcription kit (4368814; Life Technologies). Quantitative PCR was then performed using TaqMan fast advanced master mix (4444557; Life Technologies) and *CYP4V2* and *GAPDH* gene TaqMan probe sets (4331182 and 4331182, respectively; Life Technologies).

### Western blot

HEK293 or ARPE19 or iPSCs-derived RPE cell pellets were resuspended in three volumes of 1xRIPA buffer and sonicated appropriately. The lysate was mixed with 4 × Laemmli Sample Buffer (1610747; Biorad) and 2-Mercaptoethanol (1:40; M6250; Sigma) and heated at 95 °C for 5 min. The lysate was loaded on 4–15% Mini-PROTEAN® TGX™ Precast Protein Gels (4561086; Biorad). Same amount of proteins for individual samples were transferred to the PVDF membrane, followed by blocking for 1 h at room temperature and incubated with rabbit anti-CYP4V2 antibody (1:500; SAB1410565; Sigma) overnight at 4 °C. After the wash, membranes were incubated with a secondary HRP conjugated anti-rabbit IgG antibody (1:5000; NA934V; GE) and developed by chemiluminescence using ECL reagents according to the manufacturer’s instructions (GERPN2232; Cytiva).

### Construction and production of recombinant AAV

The plasmids include a kanamycin resistance gene and a 2.1 kb random stuffer sequence to reduce reverse packaging of non-transgene from the plasmid backbone (a gift from Professor Robert MacLaren, University of Oxford). Recombinant AAV2.wtCYP4V2, AAV2.coCYP4V2 and AAV2.GFP were produced in HEK293D cells packaging p.wtCYP4V2, p.coCYP4V2 (the construct shares the backbone of p.wtCYP4V2 with codon-optimised cDNA of CYP4V2, generated by GenScript), and pENN.AAV.CB7.CI.eGFP.WPRE.rBG (Addgene plasmid no. 105542) and pseudoserotyped with the AAV2 capsid (pXX2) as previously described^24^. Viral vectors were purified by AAVpro® Purification Kit (6232; Clontech Laboratories), and vector genomes were titred by real-time quantitative PCR using the following primer sets: (p.wtCYP4V2 or p.coCYP4V2-F: CAGCCATCTGTTGTTTGCCC, p.wtCYP4V2 or p.coCYP4V2-R: GGAAAGGACAGTGGGAGTGG, pENN.AAV.CB7.CI.eGFP.WPRE.rBG-F: GACGGCAACTACAAGACCCG, pENN.AAV.CB7.CI.eGFP.WPRE.rBG-R: TTCAGCTCGATGCGGTTCAC) with SYBR Green Master Mix (4309155; Life Technologies).

### Multiplicity of infection (MOI) and transduction of cells

To optimise transduction efficacy, cell lines were treated with hydroxyurea prior to AAV transduction as previously described^25^. HEK293 cells or ARPE19 cells were seeded at 2.5 × 10^5^/well in a 6-well plate and treated with 200 nM or 10 µM of hydroxyurea prior to infection with AAV2.wtCYP4V2 or AAV2.coCYP4V2 at MOI of 10^3^ vg/cell, 10^4^ vg/cell and 10^5^ vg/cell. iPS-derived RPE cells were treated with 10 µM of hydroxyurea prior to infection with AAV2.wtCYP4V2 or AAV2.coCYP4V2 at MOI of 10^5^ vg/cell. Cells were harvested 7 days post-transduction and processed for further analysis.

### Luciferin-Multi-CYP assay and cell viability assay

The luciferin-based nonselective cytochrome P450 enzyme (Multi-CYP, Promega) assay represents an indirect measurement of CYP4V2 enzyme activity as Multi-CYP (methyl 2-(6-methoxybenzo[d]thiazol-2-yl)-4,5-dihydrothiazole-4-carboxylate) is a non-selective promiscuous substrate that can be converted to D-luciferin ester by multiple P450 enzymes. D-luciferin ester is subsequently detected by Luciferin detection reagent, thus, measuring total CYP activity. Cells were seeded at 1 × 10^4^/well in a 96-well plate. Three to 7 days post-treatment, cells were replenished with 50µL of fresh medium and supplemented with 100 nM of MultiCYP substrate (P1731; Promega) for incubation at 37 °C for 3 h. 25 µL of medium was transferred to a 96-well opaque white luminometer plate and mixed with 25 μL of Luciferin Detection Reagent (V8930; Promega) to initiate a luminescent reaction. To normalize luciferin-MultiCYP assay values to cell numbers, the CellTiter-Glo® Luminescent Cell Viability Assay (G9241; Promega) was performed according to manufacturer’s instructions. Briefly, 25 μL of the CellTiter-Glo® Reagent was added to the 25 μL of remaining medium in the wells of luciferin-MultiCYP assay to initiate a luminescent reaction. 40 μL of cell lysate from each well was loaded to a 96-well opaque white luminometer plate to measure luminescence using a Spark 20 M microplate reader (Tecan).

**Figure 1:**
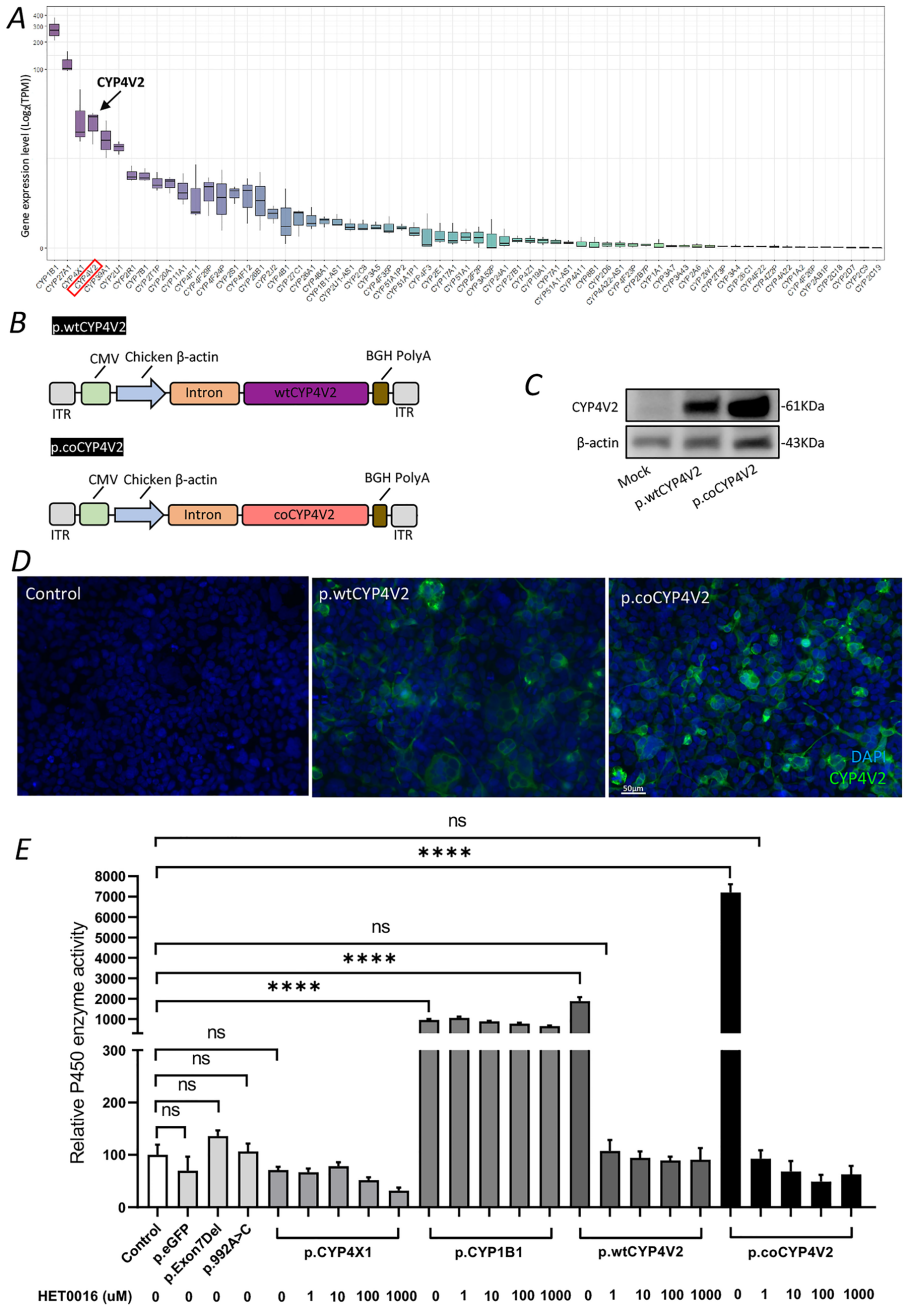
CYP4V2 expression in human RPE and plasmids *CYP4V2-*encoding plasmids. (**A**) *CYP4V2* is highly expressed in human RPE cells (TPM: transcripts per million) and ranks 4th among other CYP family genes, as determined by RNA-seq. (**B**) Schematic diagrams of plasmids encoding wild-type or codon-optimized *CYP4V2* transgenes driven by a CAG (CMV-enhanced chicken beta-actin) promoter. (**C** and **D**) Strong CYP4V2 expression was observed in HEK293 cells transfected with p.coCYP4V2 compared to those transfected with p.wtCYP4V2, assessed by western blot and immunocytochemistry analysis, respectively. (**E**) CYP enzyme activity was measured in HEK293 cells transfected with p.eGFP, p.Exon7Del, p.992A > C p.CYP4X1, p.CYP1B1, p.wtCYP4V2 and p.coCYP4V2. The latter four plasmid transfections were exposed to a dose response experiment with HET0016, a selective inhibitor of CYP4V2. Data are presented as the mean ± SEM (n = 6). ****p < 0.00001. ns, not significant.

To test the specificity of the assay, we generated two plasmids that share the construct backbone as p.wtCYP4V2, which encoded *CYP4X1* (p.wtCYP4X1) and *CYP1B1* (p.wtCYP1B1), two of the highly expressed CYP genes in human RPE cells (Fig. 1A). Moreover, to further validate the specificity of the assay and quantify the effects of two common human pathogenic *CYP4V2* mutations on this functional assay, we generated two plasmids sharing the backbone of p.wtCYP4V2 and incorporating two common CYP4V2 mutations: c.802-8_810del17insGC (which causes exon 7 to be deleted; p.Exon7Del) and c.992A > C (p.992A > C). These plasmids, along with p.wtCYP4V2 or p.coCYP4V2, were used to transfect HEK293 cells and treated with an inhibitor of CYP4V2, HET0016,^7^ at various doses, prior to the measurement of the total P450 enzyme activity by this assay.

### Human retina and RPE/choroid explant culture and AAV transduction

A healthy eye was removed from a 74-year-old patient undergoing orbital exenteration for an orbital tumour. The participant consented to his eye being utilised for this research. The eye was dissected on ice within an hour of exenteration, as previously described^26^. Briefly, the eyes were dissected to remove the iris, the lens and the vitreous, followed by cutting the sclera into four quadrants. 4 mm diameter discs of neuroretina and RPE/choroid were trephined from the surrounding tissues using a Biopsy Punch (Fig. 5A). Both retinal and RPE/choroid explants were transferred to the inserts of a 24-well transwell plates (#353095, 0.4 μm pore size, Thermo Fisher Scientific) with culture medium of neurobasal-A with phenol red supplemented with 1% penicillin/streptomycin (#15140122; Gibco), 0.8 mM L-glutamine (#25030024, Thermo Fisher Scientific), 2% 50 × B27 supplement (#17504044, Thermo Fisher Scientific), and 1% 100 × N2 supplement (#17502048, Thermo Fisher Scientific). The volume of the medium was 400 µL in the insert and 900 µL in well. Neural retina and RPE/choroid explants were cultured in the same well for 24 h at 37 °C in 5% CO_2_ incubator before AAV transduction (1 × 10^11^ vg/well). Medium was changed every 2 days. Neuroretina and RPE/choroid explants were individually processed for immunostaining.

### Statistics

All statistical analyses were performed with Prism 7 software (GraphPad Software, Inc., La Jolla, CA, USA) using student t-test for unpaired data or one-way ANOVA wherever appropriate followed by Tukey’s or Bonferroni's multiple comparisons test. The results are represented as the mean ± s.e.m. unless noted otherwise. Unless otherwise specified, a p value less than 0.05 was considered statistically significant.

## Results

### CYP4V2 expression in human RPE cells and validation of Multi-CYP functional assay

Cytochrome P450 gene expression profiling in human RPE/choroid layers using RNA-Seq (Table S1) showed that *CYP4V2* expression (transcripts per million: 25.14) ranked fourth among 65 identified P450 genes, suggesting it is enriched in human RPE/choroid layers (Fig. 1A). Plasmids encoding wild type (p.wtCYP4V2) or codon-optimised (p.coCYP4V2) human *CYP4V2* under the control of a hybrid cytomegalovirus enhancer-chicken beta actin promoter (Fig. 1B) showed greater expression of CYP4V2 in HEK293 cells transfected with p.coCYP4V2 compared with those transfected with p.wtCYP4V2 (Fig. 1C and D, Fig. S1), indicating that codon-optimised CYP4V2 transgene more effectively increased CYP4V2 protein expression in vitro compared to the wild type *CYP4V2* transgene. Notably, there was very faint detection of endogenous CYP4V2 expression in HEK293 cells by western blot, but no clear endogenous CYP4V2 expression was detected by immunocytochemistry.

To assess the functional efficacy of CYP4V2 transfections and transductions, we validated the use of a commercially available luciferase-based assay of nonselective cytochrome P450 enzyme (Multi-CYP) function in order to indirectly assess CYP4V2 enzyme activity in transfected HEK293 cells. To determine whether the Multi-CYP assay could be used to selectively isolate changes in CYP4V2 function, we first compared HEK293 cells transfected with either p.wtCYP4V2, p.CYP1B1 or p.CYP4X1 in the same plasmid backbone (the latter two CYP genes being highly expressed in human RPE cells, Fig. 1A) and exposed each group to a potent and selective cytochrome P450 inhibitor (HET0016)^27^, which is known to inhibit CYP4V2 activity^7^. Compared to untransfected and p.eGFP-transfected controls, gains in P450 enzyme activity were seen in p.wtCYP4V2- and p.coCYP4V2-transduced cells, which were also entirely inhibited by exposure to HET0016 compared to p.CYP1B1 and p.CYP4X1 (Fig. 1E). Specifically, there were no increase in CYP450 enzyme activity in cells transfected with p.CYP4X1, and exposure to HET0016 did not affect CYP450 enzyme activity in cells transfected with p.CYP1B1. Together, this suggested that the increase in Multi-CYP signal following CYP4V2 transfection was at least in large part due to exogenous CYP4V2 activity, rather than as a result of secondary changes in the expression of other cytochromes. Similarly, P450 enzyme activity was significantly increased in cells transfected with p.coCYP4V2 compared to both wild type and control cells (Fig. 1E). To determine whether the Multi-CYP assay could detected functional changes in known *CYP4V2* pathogenic variants, we used the same expression plasmid backbone to transfect HEK293 cells with one of either p.Exon7Del or p.992A > C, two common pathogenic variants (Fig. 1E). This resulted in diminished CYP4V2 protein expression compared to wild-type and codon-optimized transgenes (Fig. S2A and B) and a relative reduction in P450 enzymatic activity on the Multi-CYP assay, which approximated the effect of HET0016 inhibition on wild-type and codon-optimised expressing cells (Fig. 1E). Together, these data indicated that the Multi-CYP assay provided a reliable determination of in vitro CYP4V2 enzyme activity.

### Expression of CYP4V2 mediated by AAV2.coCYP4V2 in cultured cells

**Figure 2:**
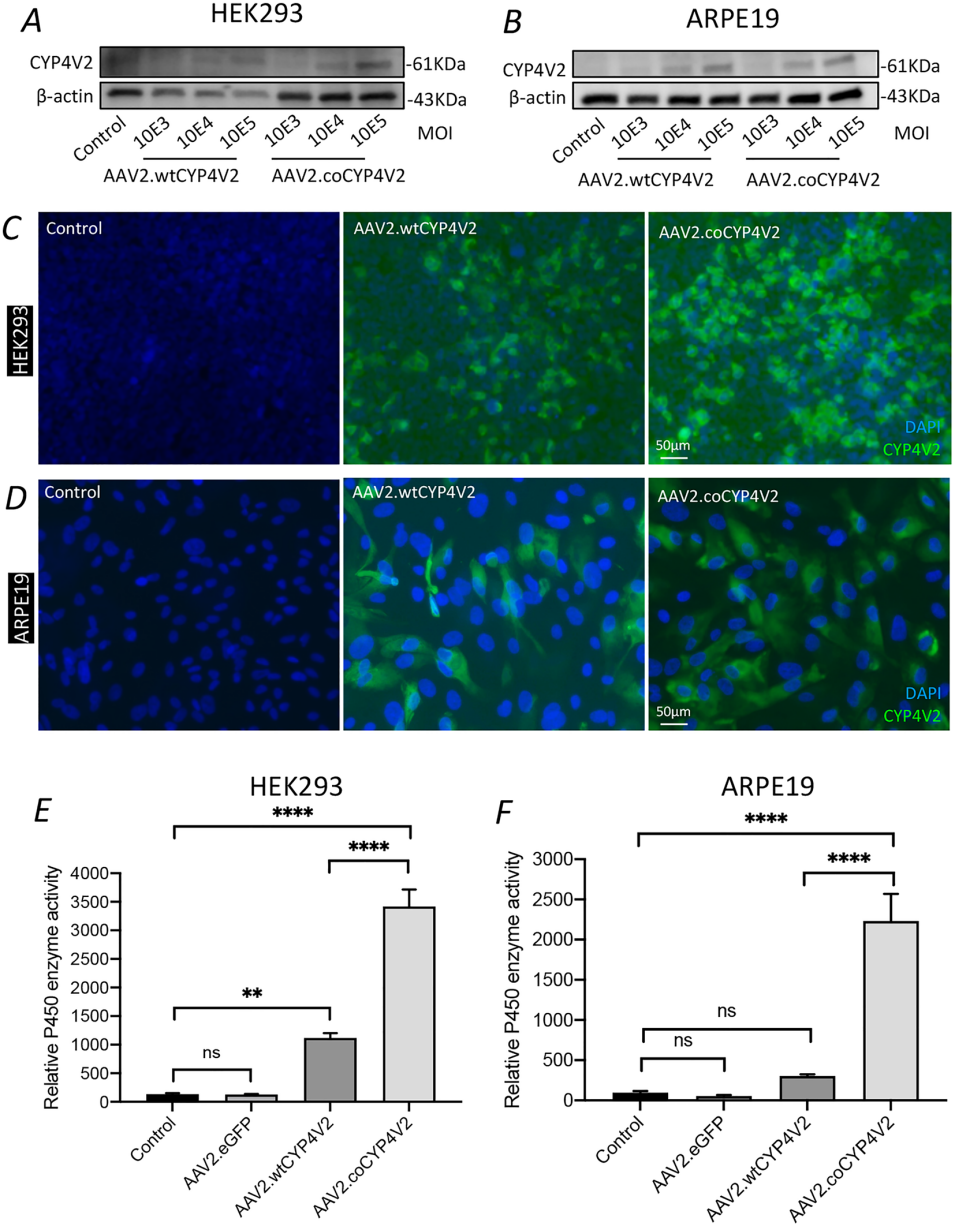
CYP4V2 expression following AAV2 transduction of cultured cells**.** (**A** and **B**) CYP4V2 expression in HEK293 and ARPE19 cells transduced with AAV2.wtCYP4V2 and AAV2.coCYP4V2, respectively, at multiplicities of infection (MOI) from 10E3 to 10E5, assessed by Western blot. (**C** and **D**) CYP4V2 expression in HEK293 and ARPE19 cells transduced with AAV2.wtCYP4V2 and AAV2.coCYP4V2, respectively at MOI of 10E5, assessed by immunocytochemistry. (**E** and **F**) CYP4V2 enzyme activity was quantified by Multi-CYP assay in HEK293 and ARPE19 cells transduced with AAV2.wtCYP4V2, AAV2.coCYP4V2 and control AAV2.eGFP. Mean ± SEM (n = 6). **p < 0.01. ****p < 0.00001. ns, not significant.

Next, we sought to establish whether codon optimisation of *CYP4V2* increased viral-mediated expression, by transducing HEK293 and human ARPE19 cells, a human retinal pigment epithelial cell line,^28^ with AAV2.wtCYP4V2 or AAV2.coCYP4V2. Western blot analysis showed that CYP4V2 protein was expressed in both HEK293 and ARPE19 cells transduced with AAV2.coCYP4V2 at MOI of 10^5^, while notable CYP4V2 expression was also observed in both cell types transduced with AAV2.wtCYP4V2 at MOI of 10^5^ (Fig. 2A and B, Fig. S3A and B). Immunocytochemistry showed that AAV2.coCYP4V2 efficiently transduced both HEK293 and ARPE19 cells, resulting in increased CYP4V2 expression in both cell types compared to AAV2.wtCYP4V2 (Fig. 2C and D). The Multi-CYP assay showed significantly elevated P450 enzyme activity in both HEK293 (~ 3.1 folds increase, *p* < 0.0001) and ARPE19 cells (~ 7.4 folds increase, *p* < 0.0001, Fig. 2E and F) transduced with AAV2.coCYP4V2 compared to AAV2.wtCYP4V2. Moreover, AAV2.coCYP4V2 transduction resulted in a MOI-dependent (10^3^–10^5^) increase in CYP4V2 functionality (Fig. S4A and B). In addition, CYP4V2 enzyme activity was significantly decreased in both AAV2.coCYP4V2-transduced HEK293 (Fig. S4C) and ARPE19 cells (Fig. S4D) when treated with the potent CYP4V2 inhibitor HET0016. Collectively, this data suggested that viral-mediated expression of wild-type, and in particular codon-optimized, *CYP4V2* increased CYP4V2 protein expression and enzymatic function in a range of cell models.

### Generation and characterization of BCD Patient-derived iPSCs

**Figure 3:**
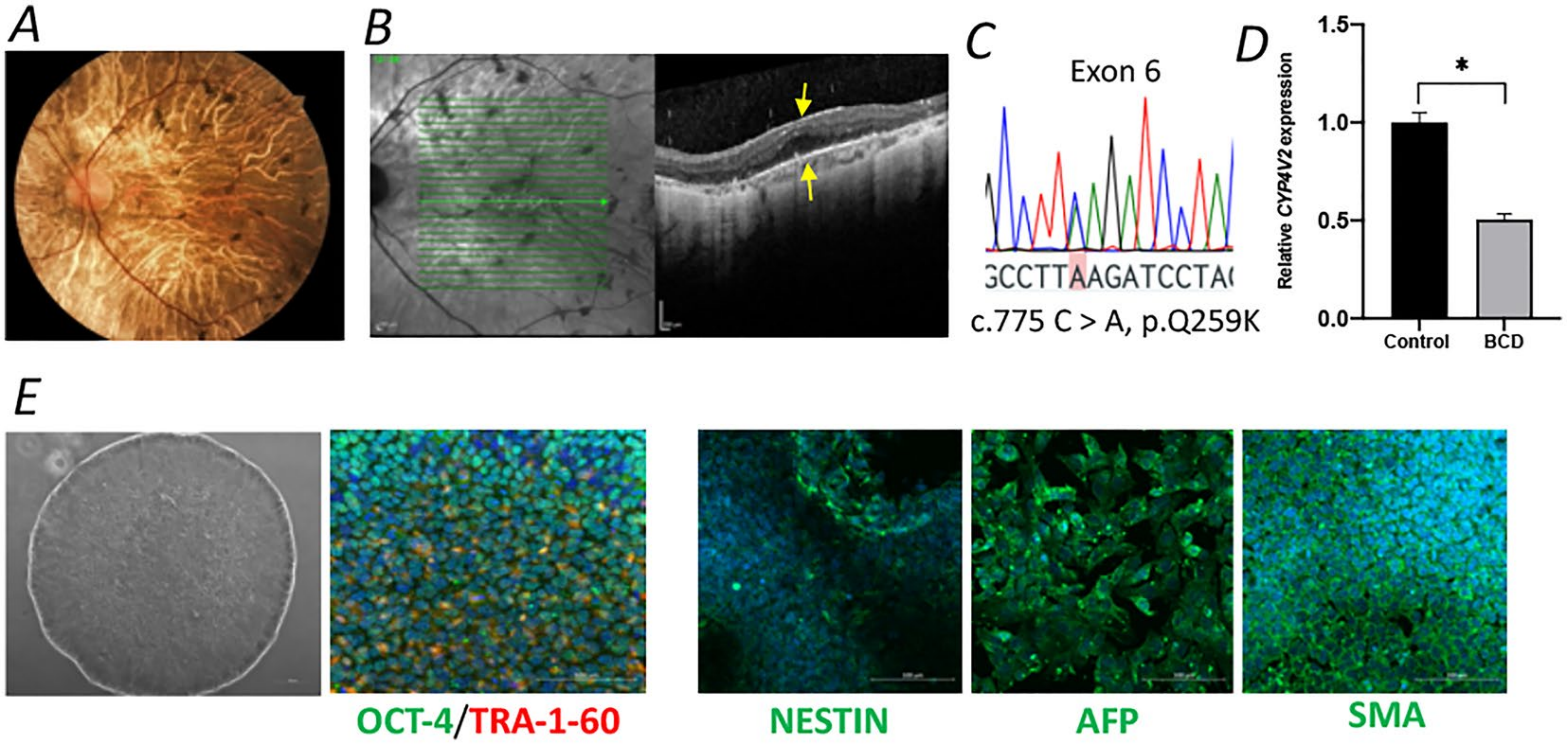
Diagnosis of a BCD patient and iPSCs generation. (**A**) Fundus image showing characteristic crystalline deposits. (**B**) OCT images show hyper-reflective crystalline deposits above the RPE in the BCD patients. Arrows indicate crystalline deposits. (**C**) Sanger sequencing showed a point mutation of *CYP4V2* in the BCD patient (highlighted in pink). (**D**) The level of *CYP4V2* mRNA in skin fibroblasts was significantly greater in the control than in the cells from the BCD patient. (**E**) Markers (including *OCT-4, TRA-1–60, NESTIN, AFP* and *SMA*) for iPSCs immunostaining. Data are presented as mean ± SEM. *p < 0.05.

To establish a human-derived disease model, iPSCs from a BCD patient were generated. On clinical examination the patient’s fundus had the crystalline deposits that are characteristic of BCD (Fig. 3A). The deposits were evident on OCT imaging as small hyper-reflective lesions in the RPE and retina (Fig. 3B). Sanger sequencing confirmed a homozygous point mutation (c.775 C > A, p.Q259K) (Fig. 3C), a known pathogenic mutation^29,30^. qPCR results from the patient’s skin fibroblasts showed that RNA expression of *CYP4V2* was significantly reduced in BCD fibroblast cells compared to control fibroblast cells (Fig. 3D). iPSCs expressed markers of pluripotency (OCT-4 and TRA-1-60), were able to differentiate into the three germ layers (assessed by embryonic body formation and expression of NESTIN, AFP and SMA, Fig. 3E).

### CYP4V2 expression in BCD patient-derived fibroblast and iPSCs-derived RPE cells

**Figure 4:**
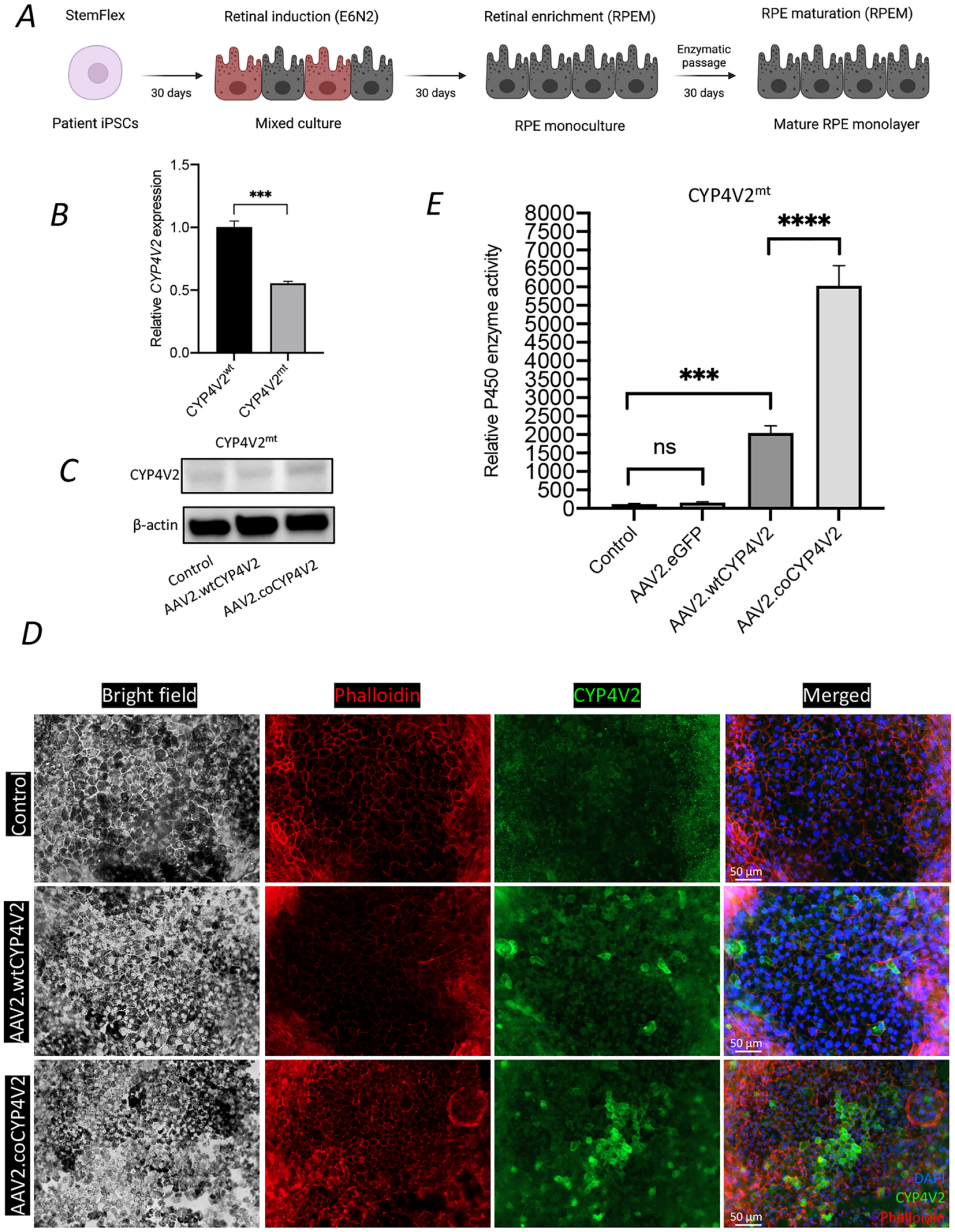
CYP4V2 expression in BCD iPSCs-derived RPE cells. (**A**) A schematic summary of the process of generation of iPSCs-RPE cells. (**B** and **C**) mRNA and protein expression of CYP4V2 was lower in BCD iPSCs-derived RPE (CYP4V2^mt^) cells than in control (CYP4V2^wt^) cells, as assessed by qPCR and western blot, respectively. (**D**) Immunocytochemistry showed stronger expression of CYP4V2 in BCD iPSCs-derived RPE cells transduced with AAV2.coCYP4V2 compared to those transduced with AAV2.wtCYP4V2 and untransduced controls. (**E**) CYP4V2 enzyme activity was quantified in BCD iPSCs-derived RPE cells transduced with AAV2.wtCYP4V2, AAV2.coCYP4V2 and control AAV2.eGFP, and data are presented as mean ± SEM (n = 12). ***p < 0.0001. ns, not significant.

BCD and wild type iPSCs were differentiated to RPE cells (CYP4V2^wt^ and CYP4V2^mt^) and were used for experimental work after 90 days (Fig. 4A)^22^. Cells demonstrated maturity by exhibiting pigmentation and cobblestone morphology^22,31^. Both lines were able to generate RPE cells in each batch of differentiation. mRNA expression of *CYP4V2* was significantly reduced in CYP4V2^mt^ RPE cells compared to CYP4V2^wt^ RPE cells (Fig. 4B), indirectly indicating the successful generation of BCD RPE cell lines. To assess if AAV2 delivery of *CYP4V2* could enhance CYP4V2 expression in the BCD RPE cells, we transduced BCD iPS-RPE cells with either AAV2.wtCYP4V2 or AAV2.coCYP4V2, followed by analysis of Western blot, immunocytochemistry, and Multi-CYP functional assay. Western blot showed modestly increased CYP4V2 protein expression in CYP4V2^mt^ RPE cells transduced with AAV2.coCYP4V2 compared to those transduced with AAV2.wtCYP4V2 (Fig. 4C, Fig. S5). Using immunocytochemistry, we observed a similar pattern of CYP4V2 expression in RPE cells transduced with AAV2.coCYP4V2 compared to those transduced with AAV2.wtCYP4V2 (Fig. 4D). A significant increase in P450 enzyme activity was observed in cells transduced with either AAV2.coCYP4V2 or AAV2.wtCYP4V2 cells compared to untransduced and AAV2.GFP transduced controls (Fig. 4E).

### AAV2-mediated CYP4V2 expression in human RPE layer ex vivo

**Figure 5:**
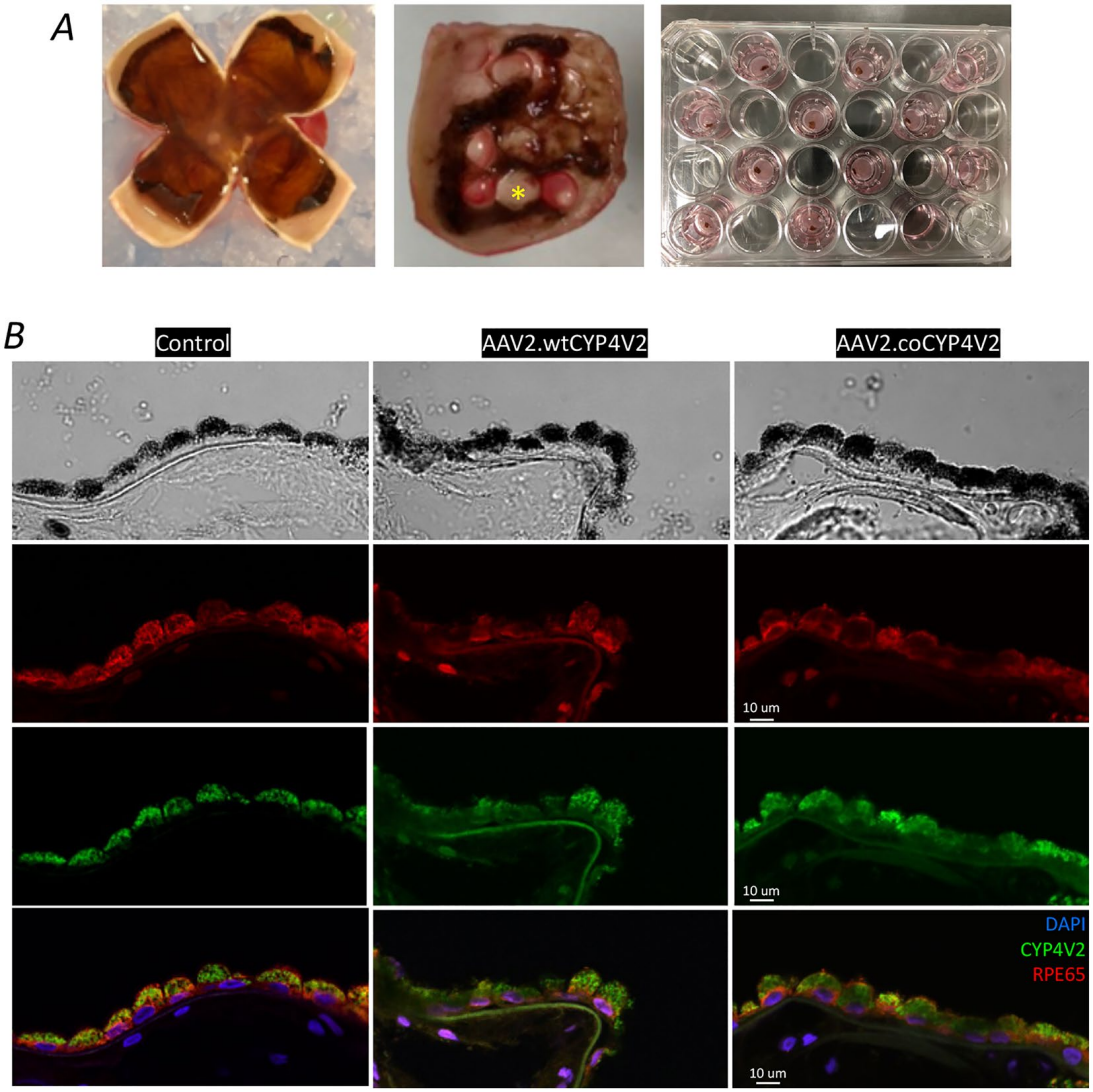
CYP4V2 expression in human RPE from retinal explant ex vivo culture transduced with AAV2. (**A**) The process of setting up ex vivo cultures of the human retina. Star indicates the 4 mm-diameter circular neural retinas and RPE/choroid trephined from the eye using a Biopsy Punch. (**B**) Immunohistochemistry showed a relatively stronger expression of CYP4V2 in the human RPE ex vivo culture transduced with AAV2.coCYP4V2 compared with those transduced with AAV2.wtCYP4V2.

To determine the AAV2.coCYP4V2 expression profile in human retina, live human neuroretina and RPE/choroid explants were transduced ex vivo (Fig. 5A). Immunohistochemistry revealed clear expression of CYP4V2 in the RPE layer of human retinal explants transduced with either AAV.coCYP4V2 or AAV.wtCYP4V2 as well as untransduced controls (Fig. 5B, Fig. S6). We did not observe CYP4V2 expression in AAV2-transduced neuroretina (Fig. S7), suggesting greater tropism for the RPE.

## Discussion

This study has demonstrated that gene therapy using both wild type (AAV2.wtCYP4V2) and codon-optimised (AAV2.coCYP4V2) *CYP4V2* vectors enhanced the level of CYP4V2 protein and function in multiple human cells, including a human iPSCs-derived RPE cells. Moreover, we showed for the first time increased CYP4V2 expression in AAV2.CYP4V2-transduced RPE cells from ex vivo human retinal cultures. Together with our validation of a functional assay that demonstrated transgene induced CYP4V2 activity, these data support a gene augmentation strategy for the treatment of BCD.

Gene therapy is beginning to show promise for the treatment of a variety of inherited ocular diseases, including the first U.S. Food and Drug Administration-approved retinal gene therapy, Luxturna® (voretigene neparvovec), for patients with IRD caused by pathological biallelic *RPE65* mutations.^32^ BCD is an ideal target for retinal gene therapy, but few studies have presented preclinical data to justify its prime candidacy for a gene augmentation strategy, partly due to the lack of well validated in vitro and in vivo models. Two recent studies have used AAV-mediated gene therapy to restore CYP4V2 functionality in the animal or cellular models^33,34^, supporting gene therapy as a feasible and effective therapeutic modality for BCD.

Codon optimization is a genetic engineering approach that applies synonymous codon substitutions to enhance transgene expression in the target cell and organism by substituting rare codons with more frequent codons and modifying the sequence to minimize destabilizing mRNA secondary structures that may inhibit translation. Codon optimisation has been widely used in preclinical gene therapy studies^35–37^, In line with previous studies, our results showed the superiority of AAV2.coCYP4V2 over AAV2.wtCYP4V2 in various human cell lines in terms of CYP4V2 protein expression and CYP4V2 enzyme activity. A moderate increase of CYP4V2 expression was also observed in ex vivo human RPE cell cultures, indicating that a potential functional rescue might be achieved by AAV2.coCYP4V2 in a clinical setting. Certainly, codon optimization may not always be superior i.e. wild type transgenes can achieve sufficient protein expression and functionality. Nonetheless, whilst codon-optimisation may not be essential for gene augmentation in BCD, it may allow equivalent transgene expression levels from lower vial titres, leading to reduced potential for local or systemic immune responses.

CYP4V2 is understood to be a microsomal fatty acid ω-hydroxylase that function on both saturated and polyunsaturated fatty acids (PUFAs) of medium and long-chain^38^. Several studies speculate that genetic defects in the catalytic function of CYP4V2 prevent local ocular degradation of lipids, resulting in its accumulation in RPE cells in patients with BCD^7,11^. The most common *CYP4V2* mutation in BCD is an insertion-deletion mutation at the junction of intron 6 and exon 7 that leads to deletion of exon 7 (c.802-8del17/insGC), resulting in significant loss of CYP4V2 enzyme activity^39^.

A study using BCD patient-derived iPSCs- derived RPE cells revealed that *CYP4V2* mutations disrupted fatty acid homeostasis, resulting in an accumulation of PUFAs, increased mitochondrial reactive oxygen species, damaged mitochondrial respiratory functions and mitochondrial stress-activated p53-independent apoptosis^33^. However, the precise substrate specificity and physiological roles of CYP4V2 remains elusive for this “orphan” P450^40^, and currently no specific assay is available to scrutinise CYP4V2 enzyme activities. We therefore validated a non-specific assay of CYP450 activity (Multi-CYP) that we utilised to indirectly assess CYP4V2 enzyme activities in cells through the non-selective proluminescent substrate. The assay could selectively detect activities of two of CYP4 enzymes we tested in HEK293 cells, including CYP1B1 and CYP4V2 but not CYP4X1, all of which are highly expressed in human RPE cells. Expectedly, we observed the significant reduction of the activity of CYP4V2 in cells transfected with plasmids encoding two of the most common pathological *CYP4V2* mutants (p.Exon7Del and p.992A > C). By using a high-affinity inhibitor of many of the human CYP4 isoforms, HET0016, that previously showed profoundly inhibitory capacity of CYP4V2^7^, we found that the Multi-CYP assay can detect the inhibitory effects induced by HET0016 in cells transfected with p.wtCYP4V2 even at a low dose (1 μM). These results suggested that the Multi-CYP assay was an effective tool for detecting changes to CYP4V2-mediated CYP450 enzyme activities in cells.

The lack of a robust disease model has made it difficult to study the mechanism by which loss of CYP4V2 function induces RPE cell degeneration in BCD patients. Although some characteristic features of corneoretinal crystal accumulation and systemic dyslipidemia were observed in a mouse model (*Cyp4v3*^*-/-*^, the murine ortholog to *CYP4V2*), the degeneration mainly occurred in photoreceptors instead of RPE cells; the tissue thought to be primarily involved in BCD^41^. Thus, studies have focussed on in vitro models, particularly iPS-derived RPE cells, to investigate the mechanism of BCD and to test therapeutic efficacy. A study using lipidomic analysis revealed the accumulation of glucosylceramide and free cholesterol in BCD-patient specific iPSCs-derived RPE cells caused RPE cell degeneration and cell death via impaired autophagy flux and lysosomal dysfunction, and BCD phenotypes in RPE cells were rescued by reducing free cholesterol by cyclodextrins or δ-tocopherol^14^. Interestingly, another study reported that excessive accumulation of PUFA led to increased mitochondrial reactive oxygen species, abnormal mitochondrial respiratory functions, thus triggering mitochondrial stress-activated p53-independent apoptosis in iPSCs-derived RPE cells derived from BCD patients^33^. Using AAV2 to deliver wild type *CYP4V2* cDNA to these mutant RPE cells, the study demonstrated that RPE cell death was rescued by reducing PUFA deposition and alleviating mitochondrial oxidative stresses in the BCD RPE cells^33^. Although having different results, both studies presented a useful in vitro model using BCD-patient specific iPSCs- derived RPE cells to study the disease. Thus, we proceeded to explore this in vitro model to obtain proof-of-concept of AAV2-mediated gene augmentation therapy for BCD. Our results showed that AAV2 encoding codon-optimised *CYP4V2* achieved the effective transduction in iPSCs-derived RPE cells from a BCD patient in terms of CYP4V2 expression and enzyme activity compared to AAV2 encoding the wild type transgene sequence. This result further confirmed rescue effects in AAV2-transduced iPSCs-derived RPE cells from BCD patents in previous reports.

Previous studies demonstrated that culturing living human retina explants is an advantageous platform for ex vivo assessment of putative human gene therapy vectors^26,42,43^. Although many effects focus on the transduction of human retina by AAV, few studies report transduction effects in human RPE cells which are the major degenerative cell types in many IRDs. Here, we showed for the first time evidence that AAV2 encoding codon-optimized *CYP4V2* effectively transduces living human RPE cells ex vivo and results in modestly enhanced expression of CYP4V2. Previous studies have shown that endogenous CYP4V2 protein is strongly expressed in both RPE layer of the healthy human retina^11^ and human iPS-derived RPE cells^14^ for which overexpression of CYP4V2 in human RPE explants might not show a clearly enhanced CYP4V2 expression. Moreover, we did not observe clear viral-mediated CYP4V2 expression in human neuroretina, suggesting wild type AAV2 is less effective for targeting the neuroretina. AAV2 is known to have higher tropism for RPE than neuroretina. A study showed that the strong transduction efficacy of AAV vectors in RPE cells compared to neural retina might be associated with the simple monolayer organization of the RPE that contrasted to multilayer structure of neural retina^44^.

A limitation of this study is that we did not examine the conformation and stability of the codon-optimized *CYP4V2* protein and compare it with the wild type-derived protein—codon changes may change sites of post-translational modification, thus potentially altering protein functions^45,46^. Secondly, we did not examine the lipid metabolic changes in cells transduced with AAV2 encoding CYP4V2 gene, although we showed evidence CYP4V2 enzyme activity was significantly increased in those cells.

## Conclusions

Using multiple cell lines and BCD-patient specific iPSC-derived RPE cells, we showed the superiority of AAV2 encoding codon-optimized *CYP4V2* against its wild-type version in terms of transgene expression and enzyme activity. We validated a CYP enzyme assay as a means of assessing CYP4V2 enzyme activity in vitro. Furthermore, we reported for the first time that wild-type AAV2 can effectively live human RPE cells ex vivo. These data provide compelling evidence for advancing CYP4V2 gene augmentation gene therapy to a clinical trial for treatment of BCD.

## Supplementary Information

Supplementary Information.
