# Supplementary material for: AAV2-mediated gene therapy for Bietti crystalline dystrophy provides functional CYP4V2 in multiple relevant cell models
Source: Sci Rep. 2022 Jun 9;12:9525. doi: 10.1038/s41598-022-12210-8 (PMC9184470; doi:10.1038/s41598-022-12210-8)
Supplement: Supplementary file 1 — Supplementary Information. [file 41598_2022_12210_MOESM1_ESM.pdf]

AAV2-mediated gene therapy for Bietti crystalline dystrophy provides functional CYP4V2 in multiple relevant cell models

Jiang-Hui Wang[1], Grace E. Lidgerwood[1,2], Maciej Daniszewski[2], Monica L Hu[1], Georgina E. Roberts[1], Raymond C.B. Wong[1,3,4], Sandy S. C. Hung[1,3], Michelle E McClements[5], Alex W. Hewitt[1,3,6], Alice Pébay[2,7], Doron G. Hickey[1], Thomas L. Edwards[1,3*]

[1]Centre for Eye Research Australia, Royal Victorian Eye and Ear Hospital, Melbourne, Australia

[2]Department of Anatomy and Physiology, The University of Melbourne, Parkville, Australia

[3]Ophthalmology, Department of

Surgery, The University of Melbourne, East Melbourne, Victoria, Australia

[4]Shenzhen Eye Hospital, Shenzhen University School of Medicine, Shenzhen, China

[5]Department of Clinical Neurosciences, Nuffield Laboratory of Ophthalmology, University of Oxford, Oxford, UK

[6]Menzies Institute for Medical Research, University of Tasmania, Hobart, TAS, Australia.

[7]Department of Surgery, Royal Melbourne Hospital, The University of Melbourne, Parkville, Australia

*Correspondence and requests for materials should be addressed to

Dr Thomas L. Edwards (tom.edwards@unimelb.edu.au). Centre for Eye Research Australia. Address: Level 7, 32 Gisborne Street, East Melbourne, VIC 3002, Australia. Tel: +61399298463.

Supplementary Figures

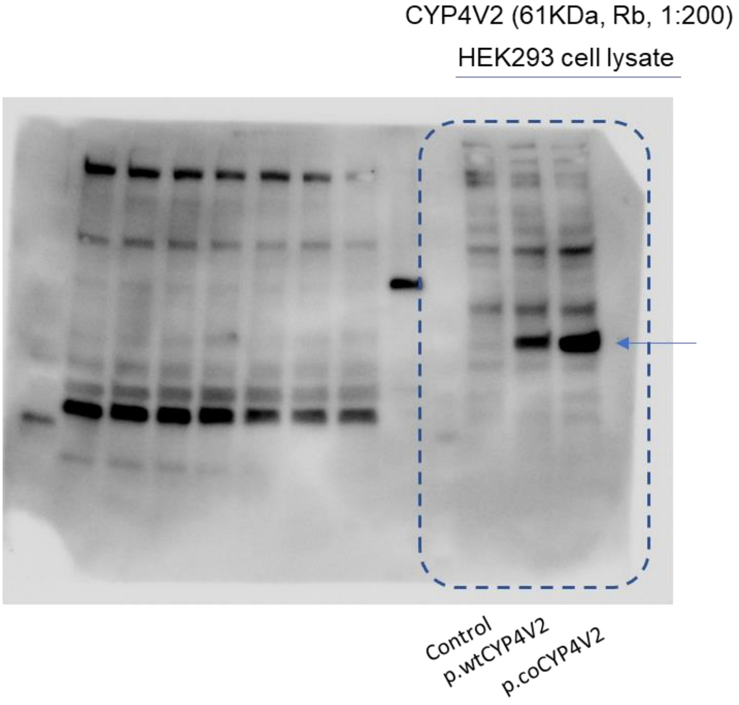

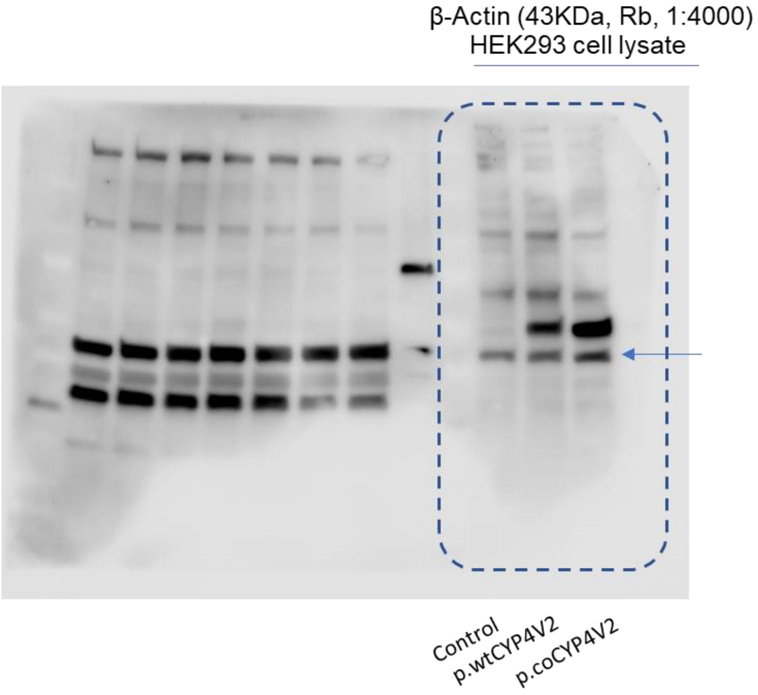

Fig. S1. Uncropped western blot images of Fig. 1C. CYP4V2 (61KDa). β-Actin (43KDa).

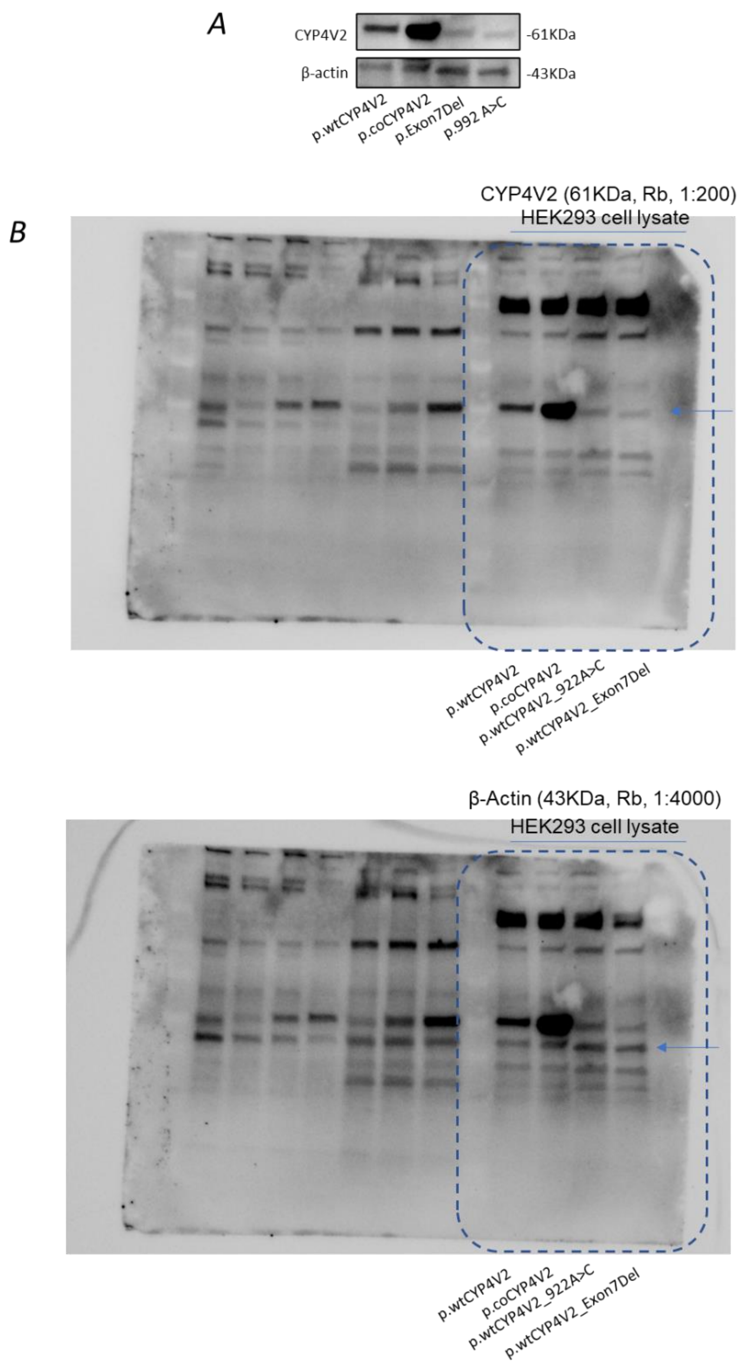

Fig. S2. Validation of the specificity of Multi-CYP functional assay in plasmid-transfected HEK293 cells. (A) Expression of CYP4V2 in HEK293 cells transfected with p.Exon7Del, p.992A>C, p.wtCYP4V2 and p.coCYP4V2, assessed by Western blot. Data are presented as mean ± SEM (n=6). ***p< 0.0001. ns, not significant. (B) Uncropped western blot images of Fig. S2A. CYP4V2 (61KDa). β-Actin (43KDa).

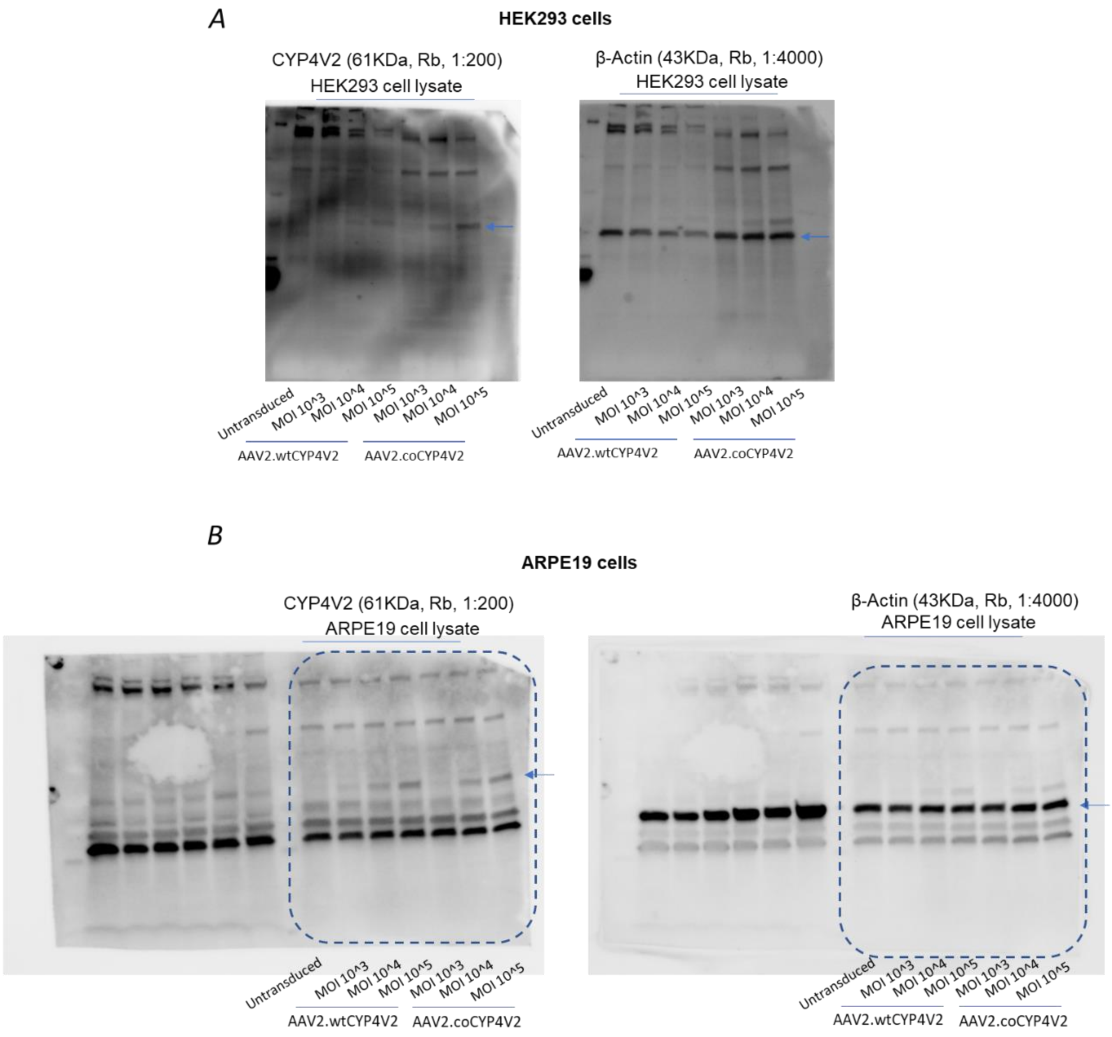

Fig. S3. Uncropped western blot images of Fig. 2A and 2B. CYP4V2 (61KDa). β-Actin (43KDa).

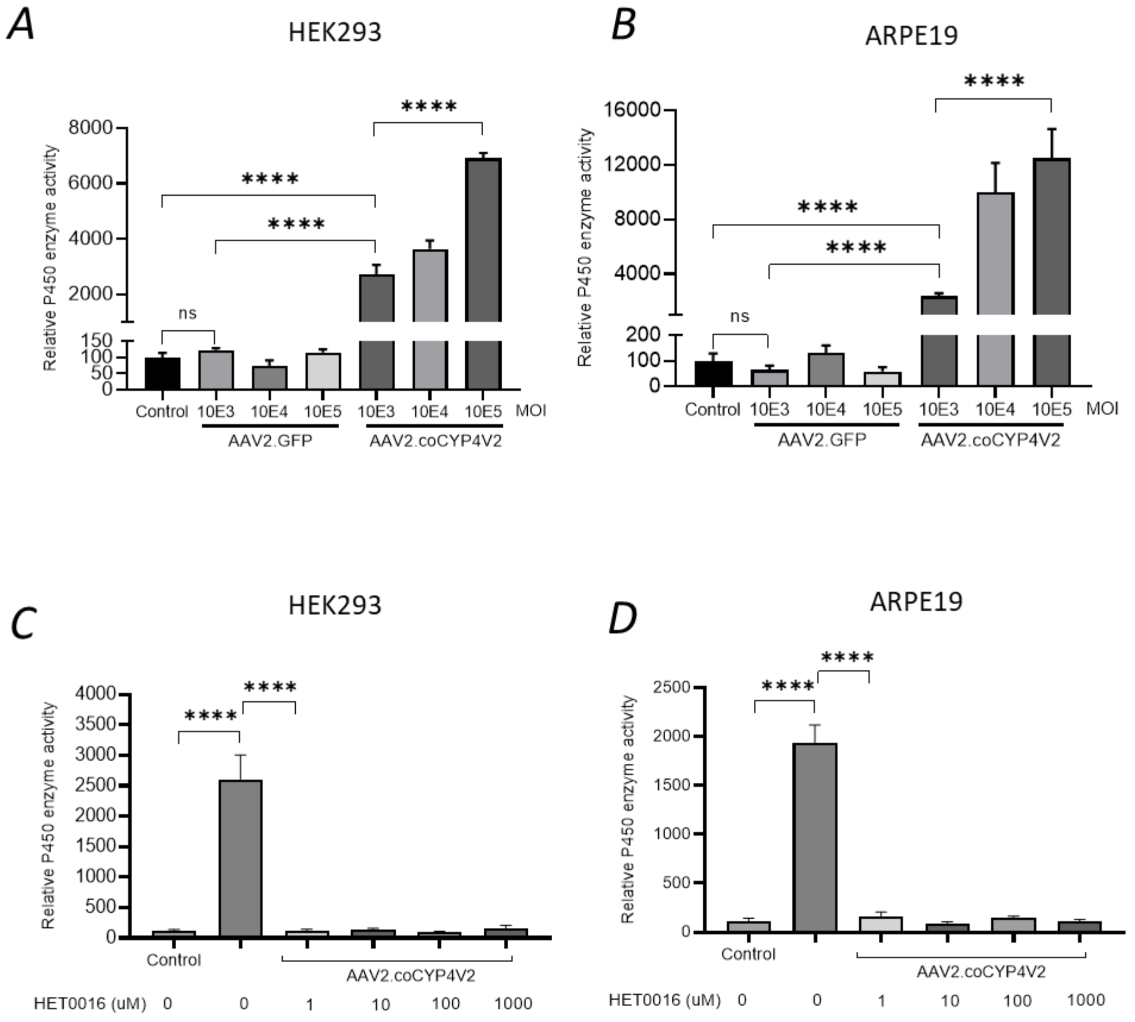

Fig. S4. Validation of the specificity of Multi-CYP functional assay in AAV-transduced HEK293 and ARPE19 cells. (A and B) CYP enzyme activity was measured in HEK293 and ARPE19 cells transduced with AAV2.GFP and AAV2.coCYP4V2, respectively, at multiple MOIs. Cells were treated with hydroxyurea overnight prior to AAV transduction. (C and D) CYP enzyme activity was measured in HEK293 and ARPE19 cells transduced with AAV2.coCYP4V2 along with HET0016 at multiple doses. Cells were treated with hydroxyurea overnight prior to AAV transduction. HU, hydroxyurea. Data are presented as mean ± SEM (n=6). ****$p < 0.00001$. ns, not significant.

iPSC-RPE cells

CYP4V2 (61KDa, Rb, 1:200)

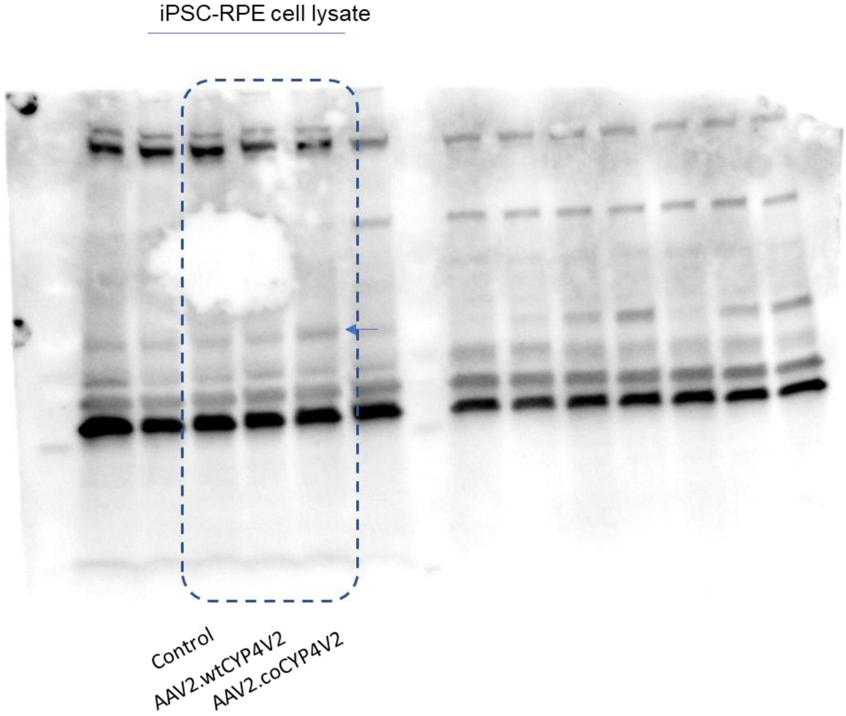

β-Actin (43KDa, Rb, 1:4000)

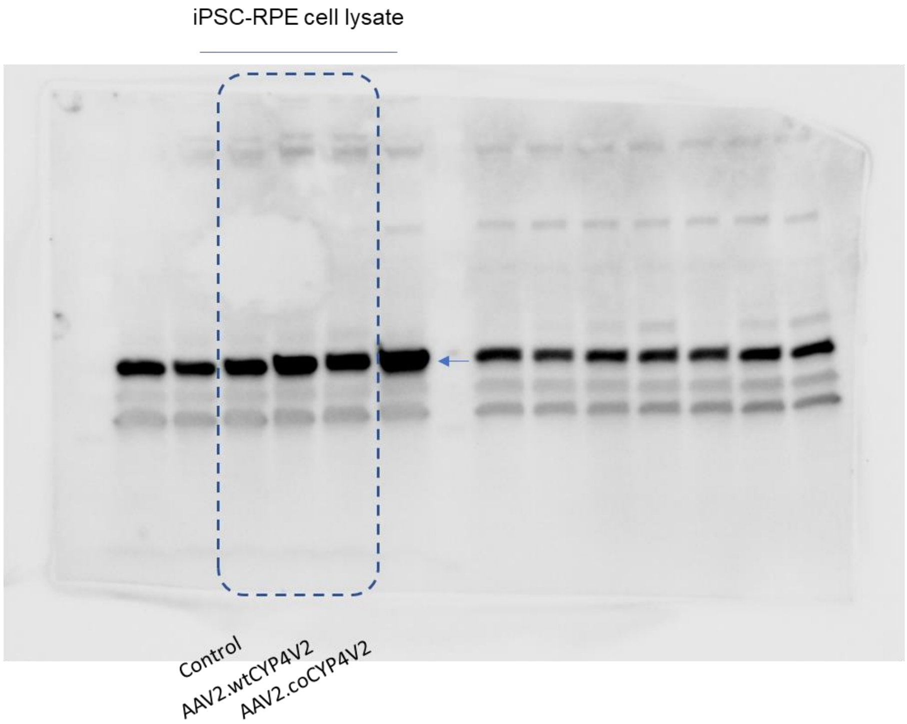

Fig. S5. Uncropped western blot images of Fig.4B. CYP4V2 (61KDa). β-Actin (43KDa).

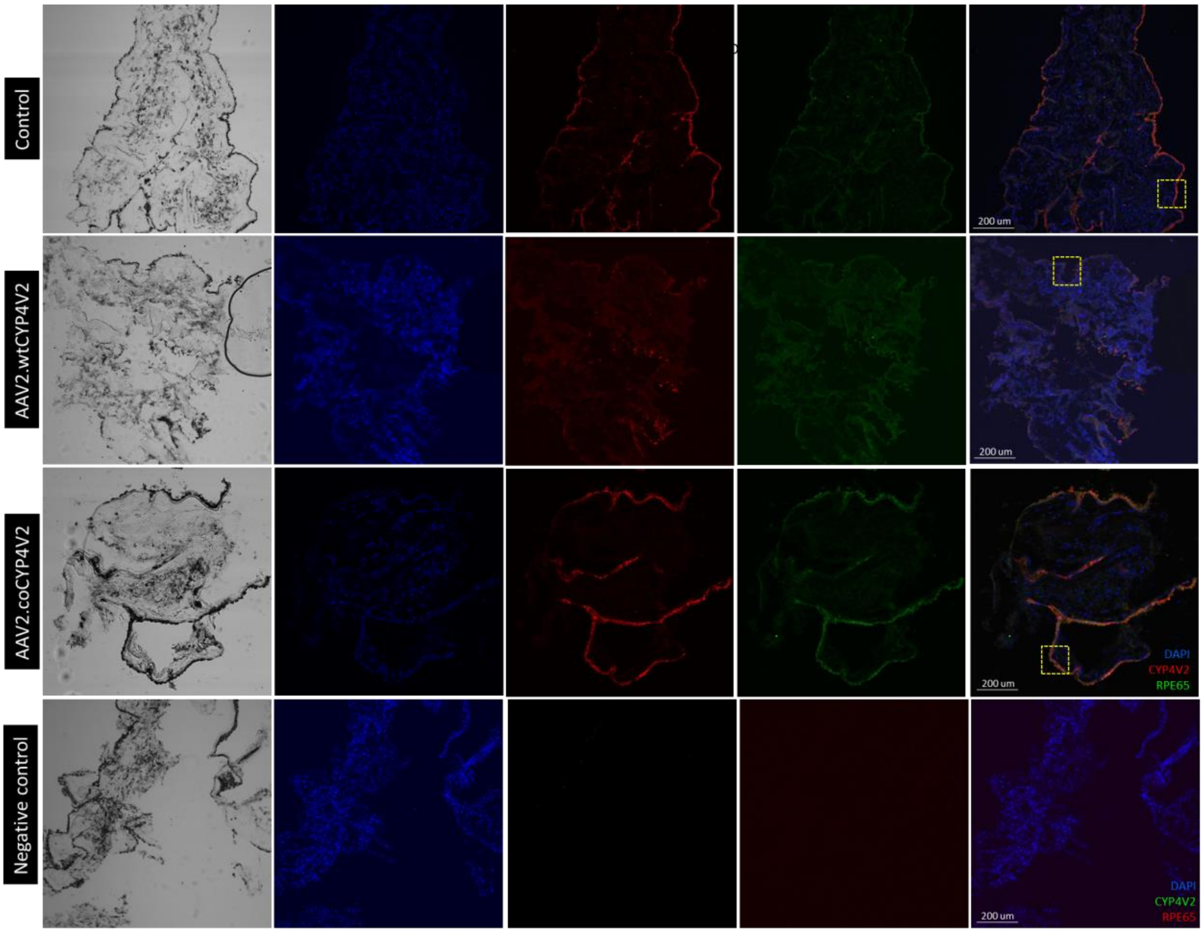

Fig. S6. Uncropped images of AAV-transduced human RPE/choroid explants. Yellow dashed square represents the area selected in Fig. 5.

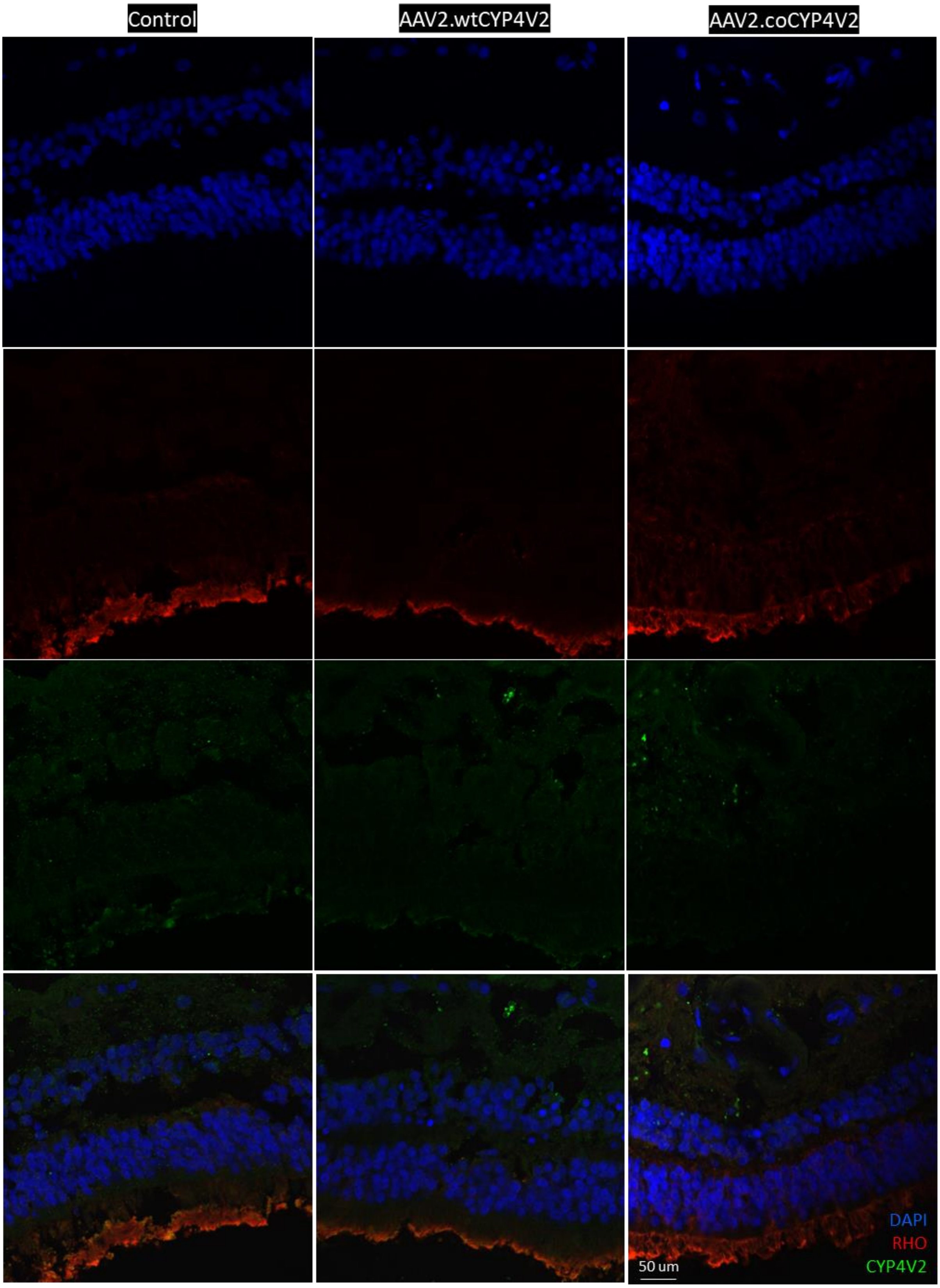

Fig. S7. CYP4V2 expression in human retinal explant *ex vivo* culture transduced with AAV2.wtCYP4V2 and AAV2.coCYP4V2.

Supplementary Tables

Table S1 Cytochrome P450 (CYP) gene expression profiling in human RPE/choroid layers using RNA-Seq. Transcriptomic data from three donors were acquired in the NCBI Gene Expression Omnibus database (GSE181550). Gene expressions were represented in transcripts per million (TPM).

GeneSymbol	SRR15358774	SRR15358775	SRR15358776	Average TPM
**CYP1B1**	371.860719	206.403804	268.376694	282.213739
**CYP27A1**	94.3673893	158.32793	101.346646	118.013989
**CYP4X1**	15.3821553	58.8044481	19.5056753	31.2307596
**CYP4V2**	29.021527	32.2288768	14.1697545	25.1400528
**CYP39A1**	15.7354948	26.2911574	9.93584235	17.3208315
**CYP2U1**	13.4712158	15.3266761	10.9536245	13.2505055
**CYP2R1**	8.18734922	5.38402265	6.02319673	6.53152287
**CYP7B1**	5.37962496	7.64686381	5.83953792	6.28867556
**CYP2T1P**	4.18367221	6.91507241	4.83075861	5.30983441
**CYP20A1**	3.82738155	5.42335521	5.98844858	5.07972845
**CYP11A1**	2.71356844	6.6654288	3.86558418	4.41486048
**CYP4F11**	8.51122304	1.87200587	2.09927952	4.16083614
**CYP4F29P**	5.93806512	1.86341192	4.6402795	4.14725218
**CYP4F24P**	0.92816375	3.37497708	7.33049405	3.87787829
**CYP2S1**	2.77994598	4.62653866	4.10598949	3.83749138
**CYP4F12**	1.3308109	4.15830636	5.76840799	3.75250841
**CYP26B1**	0.93803735	6.93397457	3.09478937	3.65560043
**CYP2J2**	2.65427508	1.35212153	2.03474328	2.0137133
**CYP4B1**	0.20148088	4.33148774	1.17064391	1.90120418
**CYP27C1**	0.5903293	2.06473881	2.25354934	1.63620581
**CYP26A1**	1.01310079	2.51870759	1.34402158	1.62527665
**CYP46A1**	1.58949424	1.77962114	1.08789436	1.48566991
**CYP1B1-AS1**	1.22910812	1.26440306	1.94413497	1.47921538
**CYP2U1-AS1**	1.08971273	0.89730827	1.62455148	1.20385749
**CYP2C8**	1.58648726	1.04631843	0.94089248	1.19123273
**CYP3A5**	1.05991216	0.96937933	1.4428232	1.15737156
**CYP4F35P**	0.75272304	1.7970751	0.89777983	1.14919265
**CYP51A1P2**	0.93173146	1.31348213	1.08270895	1.10930751
**CYP51A1P1**	0.55607118	1.41538521	0.7832446	0.91823367
**CYP4F3**	0.08860484	0.14870021	2.01913168	0.75214558
**CYP2E1**	0.26853368	0.42630381	1.38772657	0.69418802
**CYP17A1**	0.37145358	0.16970016	1.37341212	0.63818862
**CYP51A1**	0.21767243	0.55792284	1.04522316	0.60693948
**CYP2F2P**	0	0.5303032	1.14448896	0.55826405
**CYP3A52P**	0	0	1.62135936	0.54045312
**CYP24A1**	0.22764106	1.02855948	0.03699692	0.43106582

**CYP27B1**	0.38276459	0.24981092	0.62899264	0.42052271
**CYP4Z1**	0.21706612	0.65572037	0.36034208	0.41104286
**CYP19A1**	0.12428798	0.55483633	0.41139974	0.36350802
**CYP7A1**	0.21845654	0.28515087	0.48719679	0.33026807
**CYP51A1-AS1**	0.40249725	0.23642047	0.25511879	0.29801217
**CYP4A11**	0.07180799	0.54832533	0.07585794	0.23199709
**CYP8B1**	0.56883835	0.03930901	0.01767414	0.20860717
**CYP2D6**	0.17867788	0.03498418	0.34605133	0.18657113
**CYP4A22-AS1**	0.37911657	0	0.16019946	0.17977201
**CYP4F23P**	0.22113133	0	0.28032369	0.16715167
**CYP2B7P**	0.08591692	0.22205142	0.18152522	0.16316452
**CYP1A1**	0.02077753	0.43935801	0.02194938	0.16069497
**CYP3A7**	0.19771595	0.04645408	0.08354683	0.10923895
**CYP3A43**	0.05296418	0.06222063	0.19582971	0.10367151
**CYP2A6**	0.06282796	0	0.23230003	0.09837599
**CYP2W1**	0.05004176	0.02939373	0.2114564	0.09696396
**CYP2T3P**	0.08547524	0.10041359	0.09029602	0.09206162
**CYP3A4**	0.08254524	0.02424288	0.15260135	0.08646316
**CYP26C1**	0.12760763	0.03747734	0.08425291	0.08311263
**CYP4F22**	0.02673556	0.09422424	0.11297374	0.07797785
**CYP4Z2P**	0	0.15401359	0.04616511	0.06672623
**CYP4A22**	0	0.12775173	0.0574398	0.06173051
**CYP1A2**	0.04459341	0.10477382	0	0.04978908
**CYP4F26P**	0.03419847	0.08035054	0.01806363	0.04420421
**CYP2AB1P**	0.07627883	0.04480497	0	0.04036127
**CYP2C18**	0	0.03551424	0.06387173	0.03312866
**CYP2D7**	0	0.02134736	0.05758927	0.02631221
**CYP2C9**	0.01720879	0	0.01817936	0.01179605
**CYP2C19**	0.01115013	0	0.011779	0.00764304
